# What role do luting cements play in zirconia crown survival?

**DOI:** 10.1038/s41432-025-01172-1

**Published:** 2025-06-07

**Authors:** Reanna Craig, Gerry McKenna

**Affiliations:** 1 King’s College London, London, United Kingdom; 2 Centre for Public Health, Belfast, United Kingdom

**Keywords:** Health care, Dentistry

## Abstract

**A Commentary on:**

**Torres C, Mailart M C, Ávila D** **et al***.*

Influence of glass ionomer-based luting cements on the clinical success of zirconia crowns: randomized clinical trial. *Oper Dent* 2025; **50:** 144–156.

**Design:**

This 24-month prospective, split-mouth randomised clinical trial compared the performance of conventional glass ionomer cement (GIC) and resin-modified glass ionomer cement (RMGIC) for luting full-coverage ceramic-fused-to-zirconia crowns.

**Participants:**

Thirty participants were enrolled; 27 attended the 12-month review and 24 were assessed at the 24-month follow-up. Inclusion criteria included adults who needed two anterior or two posterior crowns. Silicone impressions were used to fabricate casts, which were scanned for CAD/CAM milling of zirconia copings. Clinical outcomes were assessed at 7 days, 12 months, and 24 months by calibrated, blinded examiners.

**Data analysis:**

The primary outcome was crown retention; whilst secondary outcomes included fracture, wear, pulpal response, patient satisfaction, plaque and bleeding indices, and marginal integrity. Assessments followed modified USPHS and FDI criteria. An intention-to-treat analysis using last observation carried forward was applied. Fisher’s Exact test compared anterior and posterior outcomes, while Kaplan–Meier estimates and log-rank tests were used to evaluate restoration survival (*p* < 0.05).

**Results:**

Success rates recorded were 93.3% for GIC and 100% for RMGIC. For anterior crowns, GIC success declined to 83.3% at two years, while RMGIC maintained a 100% success rate throughout. Posterior crowns showed 100% success in both groups, with no loss of retention or secondary caries recorded.

**Conclusions:**

Both GIC and RMGIC demonstrated favourable short-term outcomes. However, anterior crowns cemented with GIC were more prone to failure, suggesting that crown location should inform cement selection. Optimising luting agent choice may improve long-term clinical success.

## GRADE Rating:



## Commentary

Zirconia offers numerous advantages as a restorative material, owing to its exceptional mechanical properties, biocompatibility, and corrosion resistance^1^. Porcelain-layered zirconia crowns, used in this study, have demonstrated comparable 10-year survival rates to porcelain-fused-to-metal crowns, with failure more often due to ceramic fracture rather than loss of retention^2^. Nevertheless, the optimal cementation protocol for zirconia remains a subject of ongoing debate^3^.

This study had a clearly defined aim, with a well-formulated research question structured using the PICO framework. Randomisation and blinding were appropriately implemented, with participants assigned via external software to produce blinded codes for each group participant. Demographic and tooth-type distributions were balanced across the groups^4^, and the statistical methodology was sound: Fisher’s exact test and Kaplan–Meier analysis, followed by the log-rank test, were used to analyse group differences and estimate survival rates. A 5% significance level was applied. The results showed no statistically significant differences between the groups (*p* = 0.16), nor between anterior subgroup comparisons (*p* = 0.017). These findings may reflect the study’s limited statistical power due to relatively small sample sizes.

The authors correctly highlight the relevance of preparation geometry and cement choice in crown retention. Two anterior crowns lost retention at 12 and 24 months respectively, despite taper angles of 13.95° and 14.31°—both within the accepted convergence range of 5°–20°. However, the study did not report convergence angles for successful cases, nor did it disclose crown location or core material, which limits interpretability.

The study outlines the chemical and mechanical differences between glass ionomer cement (GIC) and resin-modified GIC (RMGIC), citing prior work by the same authors which found favourable outcomes with zirconia crowns across different core substrates. However, these variables were not directly compared in that publication. The discussion appropriately notes the limited benefit of fluoride release from these cements in preventing secondary caries, a multifactorial condition. Although no secondary caries was reported, detection is challenging without radiographic imaging or restoration removal due to zirconia’s radiopacity.

Several limitations constrain the generalisability of the findings. The single-centre design and use of convenience sampling introduce potential selection bias. Important confounding factors—such as oral hygiene status, clinician experience were not adequately controlled. The small sample size was based on a previous study that lacked a power calculation, further limiting the reliability of the findings as the study is likely underpowered. Additionally, the use of the ‘last observation carried forward’ method to manage dropout may introduce bias by assuming stability in outcomes after withdrawal, increasing the risk of a Type I error^5^. Moreover, the relatively short follow-up period limits conclusions on long-term performance.

This study reinforces the short-term viability of GIC and RMGIC as luting agents for zirconia crowns, supporting their use as affordable and conventional options in clinical practice. Longer-term, multicentre studies with broader inclusion criteria are needed to guide best practice in zirconia crown cementation.

Practice Points

- GIC and RMGIC demonstrated acceptable retention rates at two years, supporting their continued use in clinical practice.
- Successful outcomes are achievable without resin cements, particularly when appropriate resistance and retention form are optimised within preparation designs.
